# Real-world use of imipenem/cilastatin/relebactam for the treatment of KPC-producing *Klebsiella pneumoniae* complex and difficult-to-treat resistance (DTR) *Pseudomonas aeruginosa* infections: a single-center preliminary experience

**DOI:** 10.3389/fmicb.2024.1432296

**Published:** 2024-07-16

**Authors:** Cristiana Leanza, Maria Teresa Mascellino, Lorenzo Volpicelli, Sara Covino, Antonio Falletta, Francesca Cancelli, Cristiana Franchi, Martina Carnevalini, Claudio M. Mastroianni, Alessandra Oliva

**Affiliations:** ^1^Department of Public Health and Infectious Diseases, Sapienza University of Rome, Rome, Italy; ^2^AOU Policlinico Umberto I, Rome, Italy; ^3^Fondazione Eleonora Lorillard Spencer Cenci, Rome, Italy

**Keywords:** imipenem-relebactam, KPC-producing *Klebsiella pneumoniae*, imipenem/cilastatin/relebactam, ceftazidime-avibactam resistance, polymicrobial infections, antimicrobial resistance, KPC variant

## Abstract

**Introduction:**

Real-life experience with imipenem/cilastatin/relebactam (IMI/REL) for the treatment of KPC-producing *Klebsiella pneumoniae* complex (KPC-Kp) and difficult-to-treat resistance (DTR) *Pseudomonas aeruginosa* (DTR-PA) infections is herein described.

**Methods:**

Adult patients with KPC-Kp or DTR-PA infections who received ≥48 h of IMI/REL were included. Clinical and microbiological outcomes were retrieved through the medical records. Primary outcome was clinical cure. Secondary outcomes included mortality from infection onset and adverse effects attributable to IMI/REL.

**Results:**

We included 10 patients with different infections caused by DTR-PA (*n* = 4), KPC-Kp [*n* = 5, of which 3 ceftazidime/avibactam-resistant (CTV-R KPC-Kp), 2 CTV susceptible (CTV-S KPC-Kp)] or both DTR-PA/KPC-Kp (*n* = 1) successfully treated with IMI/REL: 3 hospital-acquired pneumonia, 1 ventilator-associated pneumonia, 2 skin and soft tissue infections, 1 osteomyelitis, 2 bloodstream infections, 1 complicated urinary tract infection. Clinical cure was achieved in all cases. No patients died and no side effect were reported.

**Discussion:**

We reported the preliminary real-life experience on the successful and safe use of IMI/REL for the treatment of KPC-Kp or DTR-PA complicated infections, including pneumonia and bone infections.

## 1 Introduction

Carbapenem-resistant *Enterobacterales* (CRE) and difficult-to-treat resistance (DTR) *Pseudomonas aeruginosa* (DTR-PA) infections constitute an arduous clinical challenge due to limited treatment options (Paul et al., 2022; Tiseo et al., 2022; IDSA, 2023). However, over the last decade, numerous efforts have been made to develop new molecules capable of overcoming antibiotic resistance mechanisms (Tompkins and Van Duin, 2021).

Imipenem-cilastatin-relebactam (IMI/REL) is the combination of imipenem/cilastatin (IMI) with relebactam (REL), a novel non-β-lactam diazabicyclooctane class A/C beta-lactamase inhibitor. The addition of REL restores the activity of IMI against IMI-resistant *Enterobacterales* and *P. aeruginosa*; in contrast, REL has no activity on class B and D beta-lactamases (Papp-Wallace et al., 2018; Smith et al., 2020).

The activity of IMI/REL is therefore directed toward multi-resistant Gram-negative and anaerobes pathogens and its characteristics make it a useful drug against CRE, especially those producing the KPC enzyme, and DTR-PA. Furthermore, the inherent activity of IMI against *Enterococcus faecalis* makes IMI/REL an attractive choice for polymicrobial infections such as intra-abdominal infections.

Based on the results of the registration trials, RESTORE-IMI 1 and 2, IMI/REL received approval by FDA in 2019 and EMA in 2020 for the treatment of complicated urinary tract (cUTI) and intra-abdominal infections (cIAI), with subsequent extension to nosocomial and ventilator-associated pneumonia (HAP/VAP, respectively) sustained by Gram-negative pathogens with limited treatment options^1^ (Motsch et al., 2020; Titov et al., 2021).

Currently available real-life experiences suggest a possible use of IMI/REL to treat infections sustained by CRE, DTR-PA and *E. faecalis*; however, actual clinical data remain scarce (Rebold et al., 2021).

Hereby, we describe our preliminary real-life experience on the successful and safe use of IMI/REL for the treatment of 10 patients with complicated infections caused by KPC-producing *Klebsiella pneumoniae* complex (KPC-Kp) or DTR-PA.

## 2 Methods

This is a single-center case series including adult hospitalized patients treated with IMI/REL for at least 48 h at an Academic University Hospital in Rome.

According to routine Hospitals’ Microbiology Laboratory protocol, bacterial pellet obtained from positive blood cultures (BCs) or isolated colonies from other biological samples (sputum or lower respiratory samples, intra-operative samples, deep swabs, urine) were used for bacterial identification by the Matrix-Assisted Laser Desorption Ionization–Time Of Flight Mass Spectrometry (MALDI-TOF MS) system (Bruker Daltonik GmbH, Bremen, Germany). Antimicrobial susceptibility testing was performed with the Vitek 2 automated system (bioMérieux, Marcy l’Etoile, France) and Microscan Walkaway (Beckman and Coulter, Brea, CA, USA) system. For IMI/REL susceptibility, the gradient strip test was used, and the results interpreted in accordance with guidelines (EUCAST, n.d.).

Infections were defined according to the CDC/NHSN criteria (National Healthcare Safety Network, n.d.). Hospital acquired/ventilator-associated pneumonia (HAP/VAP) were defined in accordance with CDC/NHSN surveillance definition of healthcare-associated infection for pneumonia with specific criteria (Centers for Disease Control and Prevention Website and National Healthcare Safety Network (NHSN), n.d.).

Clinical cure was defined as the resolution of symptoms and/or improvement of laboratory testing after discontinuation of antibiotic therapy.

The study was conducted in accordance with the principles of the Declaration of Helsinki and approved by the local Ethics Committee (0341/2023). The clinical and diagnostic management of the patients was carried out according to normal clinical practice.

## 3 Cases description

Ten patients were treated with IMI/REL, with a median age (interquartile range [IQR]) of 64 (57.75–71.5) years, six were males and four females. Monomicrobial infections were caused by DTR-PA (*n* = 4) and KPC-Kp (*n* = 5) [of which 3 ceftazidime/avibactam resistant (CTV-R KPC-Kp), 2 CTV-susceptible (CTV-S KPC-Kp)] and the single polymicrobial infection was caused by both DTR-PA and CTV-R KPC-Kp (*n* = 1).

Infection types included HAP (*n* = 3), VAP (*n* = 1), skin and soft tissue (*n* = 2), bloodstream infection (BSI) (*n* = 2), complicated urinary tract infection (c-UTI) (*n* = 1) and bone infection (*n* = 1). Clinical cure was achieved in all cases. No patients died and there were no side effects (Table 1).

**Table 1:** Characteristics of infections treated with imipenem/cilastatin/relebactam (IMI/REL).

Case, age and gender	Clinical history	Previous antibiotic therapy	Ward	Type of infection	Pathogen(s)	Polymicrobial	Dosage	Duration of IMI/REL therapy	Susceptibility testing of IMI/REL	IMI/REL monotherapy/combination	Outcome
Case #1	Peripheral artery disease, partial amputation of second toe of the right foot	No	Medical	SSTI of the right foot plus OM of the second toe	DTR-PA	No	1.25 g q6 h	6 weeks (plus surgical debridement)	NP	Monotherapy	Clinical cure
Case #2	Recent tooth extraction, mandibular abscess caused by *S. anginosus* and soft tissue emphysema of face and neck requiring endotracheal intubation	CTR, CLI	ICU	VAP	DTR-PA	No	1.25 g q6 h	7 days	NP	Monotherapy	Clinical cure
Case #3	Vascular ulcer left leg, multiple antibiotic allergies, chronic kidney failure	No	Medical	SSTI	DTR-PA	No	0.5 q6h	14 days	NP	Monotherapy	Clinical cure
Case #4	Chronic lymphatic leukemia, epidermolysis bullosa	FDC, DAP, AMP, FOF	Medical	BSI	DTR-PA	No	0.5 g q6 h	10 days	Susceptible (MIC 1 μg/mL)	Monotherapy	Clinical cure
Case #5	H1N1 influenza-related pneumonia with bacterial superinfection by *S. pneumoniae* with type II respiratory failure; previous bacteremic VAP by KPC-Kp	CTR, CTV, FOF, MEV	Medical	HAP	CTV-R KPC-Kp	No	1.25 g q6 h	10 days	NP	Monotherapy	Clinical cure
Case #6	COVID-19 pneumonia, Legionnaires’ disease, previous HAP by KPC-Kp, PWID	PIT, AZI, CTV, FOF	Medical	Bacteremic HAP	CTV-R KPC-Kp	No	1.25 g q6 h	14 days	Susceptible (MIC 0.38 μg/mL)	Monotherapy	Clinical cure
Case #7	Infective endocarditis and aortic valve replacement	AMP, CTR, DAP, VAN	Surgical	HAP	CTV-R KPC-Kp	No	0.75 g q6 h then 1 g q6 h	7 days	Susceptible (MIC 2 μg/mL)	Monotherapy	Clinical cure
Case #8	Renal transplant for polycystic kidney disease with liver involvement	CTV	Medical	C-UTI, hepatic abscess	CTV-S KPC-Kp (CTV MIC 8 μg/mL)	No	0.5 g q6 h	3 weeks	NP	Monotherapy	Clinical cure
Case #9	Skullcap custom prosthesis infection and empyema	MEV	ICU	BSI	CTV-S KPC-Kp	No	1.25 g q6 h	10 days	Susceptible (MIC 0.38 μg/mL)	Monotherapy	Clinical cure
Case #10	Polymicrobial necrotizing fasciitis of right leg, type 2 diabetes	FDC, TIG, COL, FOF, DAP, AMS	Surgical	OM of lower right leg and foot	CTV-R KPC-Kp DTR-PA	Yes	1.25 g q6 h	7 days (plus amputation of the lower right leg)	Susceptible (KPC-Kp MIC 1 μg/mL, DTR-PA MIC 2 μg/mL)	Combination therapy (FOF)	Clinical cure

AFib, Atrial fibrillation; AMP, ampicillin; AMS, ampicillin/sulbactam; BSI, bloodstream infection; AZI, azithromycin; FDC, cediferocol; CLI, clindamycin; COL, colistin; COPD, chronic obstructive pulmonary disease; CTR, ceftriaxone; c-UTI, complicated urinary tract infection; CTV, ceftazidime-avibactam; DAP, daptomycin; DTR-PA, difficult-to-treat *Pseudomonas aeruginosa*; FOF, fosfomycin; HAP, hospital-acquired pneumonia; KPC-Kp, *Klebsiella pneumoniae* carbapenemase-producing *Klebsiella pneumoniae* complex; ICU, intensive care unit; MEV, meropenem-vaborbactam; MIC, Minimum Inhibitory Concentration; OM, osteomyelitis; NP, not performed; PIT, piperacillin/tazobactam; PMK, pacemaker; PWID, people who inject drugs; SSTI, skin and soft tissue infection; TIG, tigecycline; CTT, ceftolozane/tazobactam; VAN, vancomycin; VAP, ventilator associated pneumonia. CTV-R, ceftazidime/avibactam resistant; CTV-S, ceftazidime/avibactam susceptible.

### 3.1 IMI/REL for the treatment of DTR-PA

#### 3.1.1 Case 1 (bone infection)

A patient with a history of peripheral artery disease and recent partial amputation involving only the distal portion of the second toe of the right foot was hospitalized due to pain, redness, and oedema of the right foot. Diagnosis of osteomyelitis (OM) of the second toe was made. She underwent debridement surgery of the second toe and the culture of intra-operative samples yielded DTR-PA, resistant to cefepime (CEP), carbapenems and ciprofloxacin. IMI/REL monotherapy was administered at a dosage of 1.25 g q6 h for 6 weeks, achieving complete remission of symptoms and clinical cure. No side effects were recorded (Table 1).

#### 3.1.2 Case 2 (VAP)

A patient with a recent tooth extraction was admitted to the ICU for a mandibular abscess caused by *Streptococcus anginosus* and soft tissue emphysema of face and neck requiring orotracheal intubation, for which she was receiving ceftriaxone (CTR). During the ICU stay, she developed VAP caused by DTR-PA. CTR was stopped and IMI/REL 1.25 g q6 h was started and continued for 7 days with symptoms remission and clinical cure. No adverse events were observed (Table 1).

#### 3.1.3 Case 3 (skin and soft tissue infection)

A patient allergic to cephalosporins with a history of chronic kidney failure was admitted to the infectious disease ward with a vascular ulcer presenting with pain, redness, and oedema of the left leg. Diagnosis of skin and soft tissue infection was made, and a deep swab of the wound showed the growth of DTR-PA, susceptible at increased exposure (I) for both imipenem (IMI-I, 4 μg/mL) and meropenem (MEM-I, 8 μg/mL). IMI/REL was therefore started at renal adjusted dosage (0.5 g q6 h) and continued for 14 days, achieving complete remission of symptoms and clinical cure and no adverse events (Table 1).

#### 3.1.4 Case 4 (BSI)

A patient with history of chronic lymphocytic leukemia was hospitalized for severe epidermolysis bullosa with polymicrobial skin and soft tissue superinfection requiring several debridements of the infected and necrotizing soft tissues of the left leg and right arm. During hospitalization, the patient acquired carbapenem-resistant *Acinetobacter baumannii* complex (CRAB) and vancomycin-resistant *E. faecalis* (VRE) rectal colonization. He underwent amputation of the right arm and had antibiotic therapy with cefiderocol (FDC) and fosfomycin (FOF) for CRAB BSI and then with ampicillin (AMP) and daptomycin (DAP) for VRE BSI. After approximately 1 month of hospitalization without need for antibiotic treatment, the patient developed fever and septic shock. Given the known colonization by multidrug resistant (MDR) organisms, empiric therapy with FDC, DAP and FOF was started. Blood cultures yielded, CTV-R, IMI-I (4 μg/mL) and MEM-I (8 μg/mL) DTR-PA (IMI/REL MIC 1 μg/mL). Antibiotic therapy was then switched to IMI/REL 0.5 g q6 h for 10 days, with early improvement and clinical cure. No side effect was recorded (Table 1).

### 3.2 Ceftazidime/avibactam-R KPC-Kp

#### 3.2.1 Case 5 (HAP)

This case describes a patient with a prolonged Intensive Care Unit (ICU) stay for severe H1N1 pneumonia complicated by *S. pneumoniae* superinfection and type 2 respiratory failure. In the ICU, he acquired rectal and respiratory colonization by KPC-Kp and further developed a bacteremic VAP due to KPC-Kp successfully treated with meropenem/vaborbactam (MEV). After transfer to the medical ward, the patient developed HAP caused by colistin and CTV-R KPC-Kp. IMI/REL was administered as monotherapy at a dose of 1.25 g q6 h for 10 days with complete remission of symptoms and clinical cure. No side effects were observed (Table 1).

#### 3.2.2 Case 6 (bacteremic HAP)

A patient was admitted to the ICU with severe COVID-19 pneumonia and Legionnaires’ disease. He was later transferred to a medical ward where he developed HAP caused by CTV-susceptible KPC-Kp, successfully treated with CTV and FOF. In the following weeks the patient had respiratory colonization by a CTV-R KPC-Kp strain and, afterwards, developed a bacteremic HAP caused by CTV-R KPC-Kp, with susceptibility to IMI/REL (MIC 0.38 μg/mL). He was then treated with IMI/REL at the dose of 1.25 g q6 h for 14 days, with early negativization of blood cultures and amelioration of respiratory gas exchanges. The patient achieved clinical cure and did not experience any side effect (Table 1).

#### 3.2.3 Case 7 (HAP)

A patient with a history of chronic obstructive pulmonary disease, atrial fibrillation, and pacemaker implantation, was hospitalized for culture-negative prosthetic aortic valve infective endocarditis and treated with AMP, CTR and DAP along with aortic valve replacement and Bentall procedure. Three days after surgery, the patient developed a CTV-R KPC-Kp HAP IMI/REL MIC 2μg/mL. Treatment with IMI/REL 0.75 g q6 h (further optimized to 1 g q6 h according to improvement of renal function) was started. After 7 days of therapy, clinical cure was achieved and IMI/REL was discontinued. No side effect was recorded (Table 1).

### 3.3 Ceftazidime/avibactam-S KPC-Kp

#### 3.3.1 Case 8 (c-UTI)

A patient with a history of renal transplantation for polycystic kidney disease and recurrent UTI associated with severe vesicoureteral reflux was discharged after an episode of C-UTI caused by KPC-Kp, successfully treated with CTV for 14 days. Three weeks later, she developed right hypochondrial pain, followed by the appearance of fever. Urine culture was positive for KPC-Kp with CTV MIC 8 μg/mL. A CT scan of the abdomen showed a 11 cm perihepatic abscess. The collection was drained radiologically, and the culture showed no bacterial growth. Because of the high CTV MIC and the presence of an intra-abdominal infection, IMI/REL 0.5 g q6 h was started, with prompt defervescence and clinical amelioration. After 3 weeks of therapy, a repeated CT scan showed the resolution of the abscess. Patient experienced no side effects and was discharged on day 25 with resolution of symptoms (Table 1).

#### 3.3.2 Case 9 (BSI)

A patient, allergic to cephalosporins and with known rectal colonization due to KPC-Kp was admitted to the ICU for a skullcap custom prosthesis infection and subdural empyema caused by KPC-Kp successfully treated with MEV and surgical debridement. Twenty-eight days after the interruption of MEV, the patient developed fever, with blood cultures showing the growth of KPC-Kp (IMI/REL MIC 0.38 μg/mL, CTV MIC 4 μg/mL, MEV MIC 1 μg/mL). Given the known allergies to cephalosporins and the unavailability of MEV at the hospital pharmacy, IMI/REL 1.25 g q6 h was administered. Blood cultures, performed after 48 h of antibiotic therapy, showed no bacterial growth. Antibiotic therapy was administered for 10 days. Clinical cure was achieved, and no side effects were recorded (Table 1).

### 3.4 DTR-PA and ceftazidime/avibactam-R KPC-Kp polymicrobial infection

#### 3.4.1 Case 10 (skin and soft tissue infection)

A patient with a history of type 2 diabetes was initially admitted to the ICU for necrotizing fasciitis of the right leg requiring repeated fasciotomies and hyperbaric chamber sessions. Intraoperative specimens identified *Streptococcus pyogenes*, *Enterobacter aerogenes*, and CRAB requiring the need of multiple courses of antibiotics within the first month of hospitalization, including FDC, tigecycline (TIG), colistin (COL), ampicillin/sulbactam (AMS), FOF and DAP. Rectal colonization by CTV-R KPC-Kp was acquired during hospital stay. Three weeks after antibiotic discontinuation, fever developed and, according to the known MDR colonizations, empirical therapy with DAP, COL, FOF and AMS was started. Deep wound microbiological samples and bone biopsy yielded CTV-R KPC-Kp (IMI/REL MIC 1 μg/mL) and DTR-PA (IMI/REL MIC 2 μg/mL). Empiric antibiotic therapy was then replaced with IMI/REL 1.25 g q6h and FOF 4 g q6 h with rapid defervescence and amelioration of the general conditions. However, the local conditions remained severely compromised, requiring lower leg amputation on day 4 of IMI/REL, which was stopped on day 7 after complete remission of systemic symptoms and in the absence of adverse events. There was no recurrence of infection at 1 month follow-up (Table 1).

## 4 Discussion

We reported a preliminary clinical experience with IMI/REL, a newly available and promising treatment option for DTR Gram-negative pathogens. This real-life case series, although with a limited sample size, gives support to the role of IMI/REL in the management of complex infections sustained by DTR-PA and KPC-Kp, the latter especially in the presence of CTV resistance or CTV higher MIC. To the best of our knowledge, our report is the first reporting the successful use of IMI/REL for the treatment of CTV-R KPC-Kp. Of note, in the cases herein described, clinical cure was always achieved, and no side effects or deaths were recorded.

The launch of new beta-lactam beta-lactamase inhibitors (BL/BLIs) such as CTV, ceftolozane/tazobactam (CTT), MEV and IMI/REL broadened the possibilities of treating infections caused by carbapenem-resistant Gram-negative bacteria (Volpicelli et al., 2021). Indeed, according to the available guidelines, the new BL/BLIs represent the drugs of choice for the treatment of infections caused by CRE (CTV, MEV and IMI/REL) and DTR-PA (CTT, CTV and IMI/REL), although the limited clinical experience with IMI/REL placed this molecule as an alternative agent (Paul et al., 2022; Tiseo et al., 2022; IDSA, 2023).

To the best of our knowledge, there are only three real-life experiences on the use of IMI/REL reported so far (Rebold et al., 2021; Larcher et al., 2022; Shields et al., 2024).

Larcher et al. carried out a monocentric observational cohort study describing a pool of hospitalized individuals who received beta-lactam antibiotics as a last resort for treating severe infections caused by DTR gram-negative bacteria. Three of them (1 HAP, 1 VAP, 1 bone and joint infection) were sustained by DTR-PA and treated with IMI/REL. Interestingly, IMI/REL was administered as monotherapy only once. Clinical and microbiological cure was obtained in all cases and one patient developed an adverse event (eosinophilia) (Larcher et al., 2022).

Encouraging results arise also from a multicenter retrospective study conducted in the U.S. including 21 infections treated with IMI/REL: 11 pneumonias (of which 2 bacteremic), 3 UTIs (of which 2 bacteremic), 3 device-associated infections (of which 1 bacteremic), 2 IAIs, 1 SSTI, 1 bone and joint infection. Infections were mostly caused by *P. aeruginosa* (16/21, 76%), even though *K. pneumoniae* was the causative agent in three patients (14%). Clinical cure was reached in 62% of cases (13/21), while the 30-day mortality was 33% (7/21). Overall, only two adverse events (AEs) occurred: 1 gastrointestinal (nausea, vomiting, diarrhea) and 1 encephalopathic effect (altered mental status, drowsiness, new-onset seizures), none of which requiring drug discontinuation. With regard to the infections specifically sustained by *K. pneumoniae* (*n* = 3), only two were caused by carbapenem-resistant strains (not specified resistance enzyme), one was Extended Spectrum Beta-Lactamase (ESBL)-producing and carbapenem susceptible. All infections were polymicrobial. Cure was achieved in two cases (66%), death occurred in two cases (Rebold et al., 2021).

The most representative experience reported so far was performed by Shields et al. in a multicenter retrospective study describing the real-world use of IMI/REL across representative US hospitals including 160 infections from 63 facilities. IMI/REL was typically administered after therapy with other β-lactams and was given for a median duration of 8 days (IQR 4–13). The most common infections were HAP or VAP (53.8%) and cUTI (16.9%). Microbiology data were available for only 37 patients, with *P. aeruginosa* being the most common (*n* = 33, 89.2%), followed by *K. pneumoniae* (*n* = 7, 18.9%), *Enterobacter cloacae* (*n* = 4, 10.8%), and *Escherichia coli* (*n* = 4, 10.8%). Polymicrobial infections occurred in 35.1% (*n* = 13) of patients. Among the *Enterobacterales*, only one was carbapenem-resistant, 10.8% (*n* = 4) were ESBL producers, while 75.7% (*n* = 28) of *P. aeruginosa* isolates were MDR. The 30-day mortality rate was 21.3% (*n* = 34) (Shields et al., 2024).

Despite the current availability of several molecules for the treatment of DTR-PA and CRE, IMI/REL presents peculiar characteristics that could make it more suitable than the other new BL/BLIs in some circumstances. In fact, both IMI and REL show good penetration in the epithelial lining fluid (ELF), corresponding to approximately 50% of plasma concentrations for each component, a value higher than CTV (26–31/35%), similar to CTT (48%) and lower than MEV (63/79%), suggesting its potential use for the treatment of lung infections (Rizk et al., 2018).

This is in accordance with the registration trials of IMI/REL, RESTORE-IMI 1 and 2. In the RESTORE-IMI 2, IMI/REL demonstrated non-inferiority to piperacillin/tazobactam (PIT) in the treatment of HAP/VAP, with promising data for both 28-day mortality (15.9% IMI/REL vs. 21.3% PIT) and favorable clinical response (61% IMI/REL vs. 55.8% PIT) (Titov et al., 2021).

Similarly, in the small HAP/VAP subgroup of the RESTORE-IMI 1 (8 treated with IMI/REL and 3 treated with COL plus IMI), which included infections caused by non-susceptible IMI pathogens, IMI/REL was associated with a good clinical response and a 20% reduction in 28-day mortality compared to the control group (Motsch et al., 2020). It should be noted, however, that in RESTORE-IMI 1 there was an overall prevalence of DTR-PA infection compared with CRE (24 vs. 6) and that the included cases were few.

In accordance to the literature (Smith et al., 2020), we found that the addition of REL to IMI was able to restore IMI *in vitro* activity in all the tested strains including both CTR-PA and KPC-Kp, as depicted in Table 2.

**Table 2:** Antibiotic susceptibility testing of KPC-producing *Klebsiella pneumoniae* complex and difficult-to-treat resistance (DTR) *Pseudomonas aeruginosa* of patients treated with imipenem/relebactam.

	Pathogen(s)	CEP (μg/mL)	PIT (μg/mL)	IMI (μg/mL)	IMI/REL (μg/mL)	MER (μg/mL)	MEV (μg/mL)	COL (μg/mL)	CTT (μg/mL)	CTV (μg/mL)
Case #1	DTR-PA	16	≥128	≥16	NP	≥16	NP	2	1	2
Case #2	DTR-PA	2	8	>16	NP	8	NP	NP	0.5	2
Case #3	DTR-PA	>8	>16	4	NP	8	NP	≤2	1	2
Case #4	DTR-PA	>8	>16	4	1	8	NP	≤2	>4	>8
Case #5	KPC-Kp	≥32	≥128	≥16	NP	≥16	NP	≥16	NA	12
Case #6	KPC-Kp	≥32	≥128	≥16	0.38	≥16	0.75	≥16	NA	≥16
Case #7	KPC-Kp	≥32	≥128	≥16	2	≥16	≥64	0.5	NA	≥16
Case #8	KPC-Kp	≥32	≥128	≥16	NP	≥16	8	≤0.5	NA	8
Case #9	KPC-Kp	>8	>16	>8	0.38	>32	1	≥2	NA	4
Case #10	DTR-PA	8	16	≥16	2	≥16	NP	≥8	1	2
	KPC-Kp	≥32	≥128	≥16	1	≥16	4	0.5	NA	12

CEP, cefepime; COL, colistin; CTT, ceftolozane/tazobactam; CTV, ceftazidime-avibactam; IMI, imipenem; IMI/REL, imipenem/relebactam; MER, meropenem; MEV, meropenem-vaborbactam; PIT, piperacillin/tazobactam; NP, not performed; NA, not applicable.

The structure of REL, which is characterized by the presence of a diazabicyclooctane core, is similar to that of avibactam (AVI); however, compared to AVI, REL has a piperidine ring which makes it more stable toward KPC-2 and prevents its efflux from the bacterial cell, lowering the probability of developing resistance (Papp-Wallace et al., 2018).

This brings to another interesting feature of IMI/REL, that is its activity toward KPC-producing strains exhibiting resistance to CTV, a profile which first emerged in 2018 and, since then, has been increasingly reported worldwide (European Centre for Disease Prevention and Control, 2018; di Bella et al., 2021; Sader et al., 2021; Shields et al., 2022; Campogiani et al., 2023; di Pilato et al., 2023; Oliva et al., 2023).

In this context, it has been recently shown that strains with KPC gene overexpression and porin alterations were able to acquire *in vitro* resistance to CTV and MEV, but still retained full susceptibility to IMI/REL, probably due to a reduced influence of OmpK36 porin mutations on IMI/REL activity (di Pilato et al., 2023).

In the three presented cases of HAP caused by KPC-Kp, with one being bacteremic, the decision to administer IMI/REL was made due to the resistance exhibited toward CTV and the unavailability of MEV in our hospital. However, treatment with IMI/REL yielded excellent clinical and microbiological responses. The same good clinical outcome was observed in the other 2 complex infections sustained by CTV-S KPC-Kp, where the decision to use IMI/REL was based on CTV high MIC and/or cephalosporin allergy. Taken together, these finding underscores the drug’s effectiveness against infections caused by KPC-Kp strains, with a special mention on those caused by strains exhibiting CTV resistance or high MIC.

Indeed, current evidence from studies such as RESTORE-IMI 2 and real-world experiences predominantly focuses on DTR-PA, with CRE infections representing only a minority of cases. Notably, in one study only three Kp-infected patients were included, of which 2 were CRE; however, no information regarding the mechanism of carbapenem resistance was available and, most importantly, they were part of polymicrobial infections (Rebold et al., 2021). In contrast, our experience involved monomicrobial KPC-Kp infections in five cases, further supporting IMI/REL’s efficacy against challenging infections sustained by this pathogen.

Interestingly, we used IMI/REL for the treatment of SSTIs and osteomyelitis caused by DTR-PA in three patients (case#3, case#10 and case#1, respectively). This pathogen is extremely common in these scenarios and clinicians often own limited therapeutic options, particularly in patients allergic to cephalosporins which are part of combinations targeted at DTR-PA, such as ceftazidime and ceftolozane. With this regard, IMI/REL showed potent *in vitro* activity against DTR-PA isolated in SSTIs worldwide (Sader et al., 2021), while, to the best of our knowledge, only two cases of bone infection successfully treated with IMI/REL have been described so far (Rebold et al., 2021; Larcher et al., 2022). Our cases add evidence toward IMI-REL efficacy in such difficult-to-treat infections, characterized by biofilm production and necessitating prolonged therapy for a successful outcome. Indeed, our experience confirmed the high tolerability of the drug, even when administered for a long period (i.e., 6 weeks).

Additionally, IMI/REL was administered as monotherapy in the majority of cases (*n* = 9) likely reflecting the clinicians’ increased confidence in using this drug alone, influenced by its favorable pharmacokinetic/pharmacodynamic (PK/PD) characteristics and its high genetic barrier to resistance.

In five cases out of 10, IMI/REL was used even without availability of *in vitro* susceptibility testing. Although the clinical and microbiological effectiveness suggested that the strains were susceptible, we strongly encourage to perform *in vitro* susceptibility test, when feasible. Indeed, despite still rare, resistance to IMI/REL has been recently reported in *P. aeruginosa* after treatment for HAP (Shields et al., 2022).

In cases #3 and #4 there was no evidence of IMI resistance but susceptibility to increasing exposure. In case #3, since the patient had history of infection relapse after previous treatment with meropenem and since the infection was in a poorly vascularized area in a patient with chronic vasculopathy, IMI/REL was preferred over IMI, and clinical cure was obtained. Likewise, in case #4, due to the evidence of resistance to CTT and CTV and the severity of clinical presentation (septic shock), the use of IMI/REL was preferred over increased-dose IMI.

Undoubtedly, there are several limitations of our report, including its retrospective nature, the small number of treated patients and the absence of IMI/REL susceptibility in all patients due to the unavailability of IMI/REL testing at our hospital at the beginning of drug use. Furthermore, whole genome sequencing and resistance determinants of DTR-PA were not performed. Nevertheless, this report details the utilization of a newly introduced drug for which there is still limited real-world experience, particularly for infections attributed to KPC-Kp. IMI/REL offers another valuable addition to the antibiotic armamentarium, especially in a country like Italy, where the prevalence of DTR organisms, including CTV-R KPC-Kp and DTR-PA, is a significant concern.

In conclusion, we demonstrated the successful use and high tolerability of IMI/REL for treating complicated infections caused by DTR-PA and KPC-Kp, especially when the latter is resistant to CTV. Despite the promising results of our preliminary report, additional prospective and multicenter studies involving all patients treated with IMI/REL are warranted in the near future.

## Data availability statement

The raw data supporting the conclusions of this article will be made available by the authors, upon motivated request.

## Ethics statement

The studies involving humans were approved by Local Hospital Ethics Committee. The study was conducted in accordance with the local legislation and institutional requirements. Written informed consent for participation was not required from the participants or the participants’ legal guardians/next of kin in accordance with the national legislation and institutional requirements due to the retrospective nature of data.

## Author contributions

CL: Conceptualization, Data curation, Investigation, Writing – original draft. MTM: Data curation, Writing – review & editing. LV: Methodology, Writing – review & editing. SC: Data curation, Writing – review & editing. AF: Data curation, Writing – review & editing. FC: Data curation, Writing – review & editing. CF: Data curation, Writing – review & editing. MC: Data curation, Writing – review & editing. CMM: Writing – review & editing. AO: Conceptualization, Supervision, Writing – review & editing.

## Footnotes

^1^https://www.ema.europa.eu/en/documents/product-information/recarbrio-epar-product-information_it.pdf
